# Are ultrasound features at the first metatarsophalangeal joint associated with clinically-assessed pain and function? A study of people with gout, asymptomatic hyperuricaemia and normouricaemia

**DOI:** 10.1186/s13047-017-0203-8

**Published:** 2017-05-22

**Authors:** Sarah Stewart, Nicola Dalbeth, Alain C. Vandal, Bruce Allen, Rhian Miranda, Keith Rome

**Affiliations:** 1 Department of Podiatry, Health & Rehabilitation Research Institute, Auckland University of Technology, Private Bag 92006, Auckland, 1142 New Zealand; 2 Faculty of Medical and Health Sciences, The University of Auckland, Private Bag 92019, Auckland, 1142 New Zealand; 3 Department of Rheumatology, Auckland District Health Board, P.O. Box 92189, Auckland, New Zealand; 4 Department of Biostatistics & Epidemiology, Faculty of Health and Environmental Sciences, Auckland University of Technology, Private Bag 92006, Auckland, 1142 New Zealand; 5 Health Intelligence & Informatics, Ko Awatea, Counties Manukau Health, Private Bag 93311, Auckland, 1640 New Zealand; 6 Horizon Radiology, Auckland University of Technology North Shore Campus, AA Building, 90 Akoranga Drive, Northcote, Auckland, New Zealand; 7 Auckland City Hospital Radiology, Auckland District Health Board, P.O Box 92189, Auckland, New Zealand

**Keywords:** Gout, Foot, Hyperuricemia, Ultrasonography

## Abstract

**Background:**

The first metatatarsophalangeal joint (1st MTP joint) is a common location for sonographic evidence of urate deposition in people with gout and asymptomatic hyperuricaemia. However, it is unclear whether these are related to clinically-assessed pain and function. This study aimed to determine the association between ultrasound features and clinical characteristics of the 1st MTP joint in people with gout, asymptomatic hyperuricaemia and age- and sex-matched normouricaemic individuals.

**Methods:**

Twenty-three people with gout, 29 with asymptomatic hyperuricaemia and 34 with normouricaemia participated in a cross-sectional study. No participant had clinical evidence of acute inflammatory arthritis at the time of assessment. Four sonographic features at the 1st MTP joint were analysed: double contour sign, tophus, bone erosion and synovitis. Clinical characteristics included in the analysis were 1st MTP joint pain, overall foot pain and disability, 1st MTP joint temperature, 1st MTP joint range of motion and gait velocity. Statistical analyses adjusted for the diagnostic group of the participant.

**Results:**

After accounting for the diagnostic group, double contour sign was associated with higher foot pain and disability scores (*P* < 0.001). Ultrasound tophus was associated with higher foot pain and disability scores (*P* < 0.001), increased temperature (*P* = 0.005), and reduced walking velocity (*P* = 0.001). No associations were observed between ultrasound synovitis or erosion and the clinical characteristics.

**Conclusions:**

Ultrasound features of urate crystal deposition, rather than soft tissue inflammation or bone erosion, are associated with clinical measures of foot-related functional impairment and disability even in the absence of clinical evidence of current acute inflammatory arthritis. This association persisted regardless of the diagnosis of the participant as having gout or asymptomatic hyperuricaemia.

## Background

Musculoskeletal ultrasonography has gained notable recognition among imaging modalities used in the assessment of gouty arthritis due to its ability to visualise not only soft tissue inflammation and joint damage, but also monosodium urate (MSU) crystals. Recent sonographic studies have demonstrated MSU deposition and inflammation within musculoskeletal structures in patients with gout even in the absence of current symptoms of acute arthritis [1–3]. Furthermore, hyperuricaemic individuals, who have never experienced an episode of acute arthritis, also demonstrate subclinical deposition of MSU crystals on ultrasound assessment [4–7]. The long-term relevance of ongoing subclinical disease activity in these individuals is uncertain.

MSU crystal deposition primarily targets peripheral structures of the lower limbs and individuals with both gout and asymptomatic hyperuricaemia present with high levels of self-reported foot pain and disability when compared to healthy people with normal urate levels [8]. Biomechanical-based research has also shown that they demonstrate lower limb functional impairments related to spatiotemporal parameters of gait and plantar foot loading [9–11].

The first metatarsophalangeal joint (1st MTP joint), which plays an important functional role during the propulsive phase of gait, is the most common joint affected in people with gout. A recent meta-analysis estimated 73% of people with gout experience symptoms of acute arthritis at the 1st MTP joint at least once during the course of their disease [12]. In addition, patients with gout report persistent 1st MTP joint pain and exhibit local structural and functional changes at this joint including reduced muscular strength, limited joint motion and an increased prevalence of musculoskeletal deformity [8].

Associations have been shown between imaging findings and clinical measures of foot pain and function in people with rheumatoid arthritis [13] and people without arthritis with painful 1st MTP joints [14]. However, it is unclear whether 1st MTP joint sonographic features are related to clinical measures of structure, function and pain in people with gout or asymptomatic hyperuricaemia, despite the important functional role of this joint and its susceptibility to MSU deposition. This study aimed to determine the association between sonographic features and clinical characteristics of the 1st MTP joint in people with gout, asymptomatic hyperuricaemia and age- and sex-matched normouricaemic individuals.

## Methods

We analysed data from a cross-sectional study examining both ultrasound features and foot function in people with gout, asymptomatic hyperuricaemia, and age- and sex-matched normouricaemic controls [1, 8, 9]. This analysis of the association between ultrasound findings and foot pain and function was pre-specified in the study protocol.

### Participants

Ethical approval for the study was obtained from the AUT Ethics Committee (13/100). All participants provided written informed consent prior to data collection. A total of 86 participants (gout *n* = 23, asymptomatic hyperuricaemia *n* = 29, and age- and sex-matched normouricaemic controls *n* = 34) were included in the study. This sample size was calculated from existing prevalence rates of the double contour sign at the 1st MTP joint [2, 5, 15–17], which provides approximately 80% power to detect a difference between asymptomatic hyperuricaemia and controls and 87% power to detect a difference between gout and controls at a significance level of 5% against two-side alternatives. Participants with gout were recruited from Auckland District Health Board, New Zealand, using a consecutive sampling technique. All participants with gout fulfilled the 1977 preliminary American Rheumatism Association (ARA) classification criteria for gout [18]. Participants without gout were recruited from Auckland University of Technology (AUT) staff and underwent serum urate capillary testing on the day of the study using a Reflotron® Plus (Roche Diagnostics Ltd., New Zealand). They were stratified into either the asymptomatic hyperuricaemic group (serum urate ≥ 6.9 mg/dL) or the normouricaemic control group (serum urate < 6.9 mgl/dL). The three groups were age-and sex-matched. Participants were excluded if they were aged under 20 years (to meet the requirements of the AUT Ethics Committee); had a history of other inflammatory arthritis; were experiencing acute arthritis at the time of the study; had foot and/or ankle surgery in the previous three months; had a history of 1st MTP joint surgery; or lower limb amputation. Demographic data were obtained from all participants including age, gender, ethnicity, body mass index (BMI), current medications and medical history. Additionally, gout disease characteristics were documented for participants with gout including disease duration, flare history and tophus presence.

### Sonographic features

The ultrasound examination was performed at the AUT Horizon Scanning Clinic by a single experienced musculoskeletal radiologist (BA) who was blinded to all clinical features including gout status and serum urate results. A Phillips iU22 diagnostic ultrasound machine (Bothell, Washington, USA) with a 10 MHz, 55 mm linear array transducer was used. All B-mode settings, including frequency, focal zones, gain and depth were standardised across participants. Bilateral 1st MTP joints were scanned with participants positioned supine with legs extended. A water-based gel was applied to the skin to optimise transducer-skin contact and to provide an acoustic interface. The dorsal, medial and plantar aspects of each joint were scanned using a multi-planar technique, in which transverse and longitudinal planes were imaged. Each joint was maximally dorsiflexed and plantarflexed by the radiologist during scanning to ensure direct visualisation of the articular surface of the first metatarsal head. A dynamic technique was adopted in which the probe insonation angle was manipulated by the sonographer to reduce the occurrence of the cartilage interface sign. Each joint was scanned in B-mode grey scale and then using power Doppler. Power Doppler involved the use of a standardised pulse repetition frequency of 400 to 500 Hz and low wall filters with the gain adjusted to a level just below the disappearance of the colour signs within the bony cortex [19, 20].

Two musculoskeletal radiologists (BA and RM) who were blinded to all clinical features, including diagnostic group status and serum urate results, and to each other’s scores, independently reviewed the static images for the presence of four ultrasound features: the double contour sign, tophus, erosion and synovitis. The double contour sign was defined as an abnormal hyperechoic band over the superficial margin of the articular cartilage, independent of insonation [21]. Tophus was defined as a circumscribed, inhomogeneous, hyperechoic and/or hypoechoic aggregation that may or may not generate a posterior acoustic shadow and that may be surrounded by a small anechoic rim [21]. Erosions were defined as intra-articular discontinuity of the bone surface, visible in two perpendicular planes [21]. Synovitis was defined as the presence of the power Doppler signal (including both single and confluent vessel signals) within the joint space [22]. For the purpose of the inferential analyses, ultrasound features were considered present only if scored as present by both readers.

### Clinical foot assessment

Both right and left 1st MTP joint pain over the past week was assessed using 100 mm Visual Analogue Scales (VAS) anchored with ‘no pain’ (0 mm) and ‘worst pain imaginable’ (100 mm). Patient-reported overall foot pain and disability was assessed using the 19-item Manchester Foot Pain and Disability Index (MFPDI) [23]. Each item was answered ‘none of the time’ (scored as 0), ‘on some days’ (scored as 1) or ‘on most/every day(s)’ (scored as 2) in the past month and a total score out of 38 was summated for each participant.

Temperature of the 1st MTP joint was measured using a DermaTemp 1001 (Exergen Corporation, Massachusetts), which is a hand-held infrared thermographic scanner with an in-built sensor. Participants were given adequate equilibration time in the room (thermostatically controlled at 22 °C ± 2 °C). Three temperature readings were recorded from the dorsal aspect of the 1st MTP joint for each foot, and the mean of the three trials calculated for use in the inferential analyses.

Passive, non-weight-bearing 1st MTP joint dorsiflexion range of motion (ROM) was measured using a hand-held goniometer (Whitehall Manufacturing Ltd., California, USA) in accordance with the procedure outlined by Hopson and McPoil [24]. Participants were positioned seated with knees extended and the ankle in neutral. Lines were drawn on the medial aspect of the foot along the sagittal bisections of the first metatarsal and proximal phalanx. The examiner applied a dorsiflexion force to the hallux until it could no longer be passively moved into further extension. The angle between the two bisection lines was measured from the goniometer. Three repeated measurements of right and left feet were taken and the mean for each foot calculated for use in the inferential analyses.

Walking velocity (m/s) during level barefoot walking was collected using the GAITRite system (CIR Systems, Inc., New Jersey, US). The GAITRite is a 700 cm x 90 cm electronic walkway with an active sensor area of 610 cm long and 60 cm wide. The active area contains 23,040 embedded pressure-activated sensors with a spatial resolution of 1.27 cm and a sampling rate of 120 Hz. All data was processed and stored by an IBM compatible computer using GAITRite® gold, Version 3.2 b software. Participants were instructed to walk at their own comfortable walking speed [25] from a point 100 cm before the walkway and finishing 100 cm past its end to ensure that when they reached the walkway they were walking at a normal speed and momentum. Three trials of barefoot walking were recorded for each participant with adequate rest time between trials and the mean of the three trials used for the inferential analyses.

### Statistical analysis

All descriptive data were described as mean (SD) for continuous data and frequency (%) for categorical data. All clinical outcome measures were reviewed for normality using the residuals from a linear model. Mixed linear regression models were used to determine the univariate associations between the sonographic features (independent variables) and clinical characteristics (dependent variables) of the 1st MTP joint. The overall models included the sonographic feature as a fixed effect as well as the interaction effect between the sonographic feature and diagnostic group. All models accounted for repeated measures taken from right and left feet through inclusion of a participant-specific random effect and participant-nested random effect for foot-side were added to the model. This analysis produces results identical to an analysis of measures averaged for each foot-side that would allow for a between-foot-side correlation and also allows for any reweighting required due to missing values. Bonferroni-adjusted significance levels were used for the inferential analyses (*P* < 0.01). Data were analysed using IBM SPSS Statistics version 22.

## Results

All participants were men with a mean age of 58 years and predominantly of European ethnicity (Table 1). Participants with gout and asymptomatic hyperuricaemia had significantly higher BMI compared to the controls (*P* < 0.05). Participants with gout had a mean (SD) disease duration of 18 (11) years. Eighty-three percent had a history of 1st MTP joint acute arthritis and 26% had clinical evidence of 1st MTP joint tophi. Descriptive statistics for the ultrasound features and clinical characteristics are also presented in Table 1.

**Table 1:** Descriptive statistics for participant, sonographic and clinical characteristics

Variable	Control	Gout	Asymptomatic hyperuricaemia
*N*	34	23	29
Gender, male, *n* (%)	34 (100)	23 (100)	29 (100)
Age, years	58 (14)	58 (14)	58 (19)
Ethnicity, *n* (%)	European 30 (88)	European 14 (61)	European 24 (83)
	Maori 1 (3)	Maori 1 (4)	Maori 0 (0)
	Pacific 0 (0)	Pacific 4 (17)	Pacific 3 (10)
	Asian 3 (9)	Asian 4 (17)	Asian 2 (7)
BMI, kg/m^2^	25.0 (2.9)	30.8 (3.8)^a^	29.3 (5.9)^a^
Diuretic use, *n* (%)	4 (12)	3 (13)	7 (24)
NSAID use, *n* (%)	7 (21)	14 (61)^a^	11 (38)
Prednisone use, *n* (%)	0 (0)	5 (22)	0 (0)
Hypertension, *n* (%)	9 (26)	16 (70)^a^	16 (55)^a^
Cardiovascular disease, n (%)	1 (3)	6 (26)^a^	5 (17)
Diabetes, *n* (%)	2 (6)	4 (17)	1 (3)
Serum urate, mg/dl	5.4 (1.0)	5.9 (1.7)	7.7 (0.8)^a^
Disease duration, years	-	18 (11)	-
Number of acute flares in preceding 3 months	-	1.4 (1.4)	-
1MTP flares in preceding 3 months, *n* (%)	-	6 (26)	-
History of 1st MTP joint flares, *n* (%)	-	19 (83)	-
Presence of clinically-evident tophi at any site, *n* (%)	-	17 (74)	-
Presence of clinically-evident tophi at the 1st MTP joint, *n* (%)^b^	-	6 (13)	-
Urate lowering therapy^b^, *n* (%)	-	22 (96)	-
Microscopically-proven gout, *n* (%)	-	6 (26)	-
US double contour sign^b^, *n* (%)	9 (13)	17 (37)	21 (36)
US tophus^b^, *n* (%)	0 (0)	6 (13)	0 (0)
US erosion^b^, *n* (%)	2 (3)	15 (33)	1 (2)
US synovitis^b^, *n* (%)	5 (7)	20 (44)	2 (3)
1st MTP joint pain VAS^b^, mm	1.6 (4.0)	8.5 (15.6)	6.9 (15.1)
Manchester Foot Pain and Disability Index	1.8 (6.0)	13.4 (10.8)	3.4 (4.8)
1st MTP joint range of motion^b^, °	77.6 (17.4)	59.0 (19.6)	76.5 (16.9)
1st MTP joint temperature^b^, °C	25.3 (2.1)	27.4 (2.8)	26.1 (2.2)
Gait velocity, m/s	1.05 (0.19)	0.88 (0.17)	1.05 (0.24)

Values presented as mean (SD) unless otherwise indicated. ^a^Significantly different from control group (*P* < 0.05). ^b^Values are based on joints (controls = 68 joints; gout = 46 joints; asymptomatic hyperuricaemia = 58 joints). *BMI* body mass index, *NSAID* non-steroidal anti-inflammatory drugs, *US* ultrasound, *VAS* Visual Analogue Scale, ^o^ degrees

The distribution of residuals from the linear models for all continuous outcome measures demonstrated sufficient normality to carry out parametric testing. The interaction effect between the sonographic feature and diagnostic group was significant (*P* < 0.05) in the majority of analyses (data not reported), indicating that associations between the ultrasound and clinical variables were different across groups. After accounting for the diagnostic group, presence of the double contour sign was significantly associated with higher MFPDI scores (*P* < 0.001) (Table 2). After accounting for the diagnostic group presence of tophus was significantly associated with higher MFPDI scores (*P* < 0.001), increased 1st MTP joint temperature (*P* = 0.005) and reduced walking velocity (*P* = 0.001) (Table 3). No associations were observed between erosion and the clinical characteristics (Table 4) or synovitis and the clinical characteristics (Table 5).

**Table 2:** Association between clinical characteristics and presence of the double contour sign

	Least squares mean		Diff.	95% CI for Diff.		*P*
	Present	Absent		Lower	Upper	
1st MTP joint Pain VAS, mm	3.4	7.0	3.5	−1.0	8.0	0.12
Manchester Foot Pain and Disability Index	10.1	4.8	−5.4	−7.8	−2.9	<0.001^a^
1st MTP joint range of motion,°	68.8	72.2	2.4	−3.2	8.1	0.40
1st MTP joint temperature, °C	26.8	26.0	−0.8	−1.5	0.1	0.018
Walking velocity, m/s	1.02	1.00	−0.02	−0.10	0.05	0.52

Results are presented adjusted for diagnostic group. ^a^Significantly different at Bonferroni-adjusted level of < 0.01. *Diff.* Difference in mean estimate between presence and absence, *CI* Confidence Interval, *1st MTP joint* first metatarsophalangeal joint, ^o^ degrees

**Table 3:** Association between clinical characteristics and presence of tophus

	Least squares mean		Diff.	95% CI for Diff.		*P*
	Present	Absent		Lower	Upper	
1st MTP joint Pain VAS, mm	2.4	6.0	3.6	−7.0	14.3	0.50
Manchester Foot Pain and Disability Index	19.8	5.9	−13.9	−19.7	−8.1	<0.001^a^
1st MTP joint range of motion, °	55.1	71.2	16.1	2.4	29.7	0.021
1st MTP joint temperature, °C	28.6	26.2	−2.3	−3.9,	−0.7	0.005^a^
Walking velocity, m/s	0.88	1.00	0.11	0.05	0.18	0.001^a^

Results are presented adjusted for diagnostic group. ^a^Significantly different at Bonferroni-adjusted level of < 0.01. *Diff.* Difference in mean estimate between presence and absence, *CI* Confidence Interval, *1st MTP joint* first metatarsophalangeal joint, ^o^ degrees

**Table 4:** Association between clinical characteristics and presence of erosion

	Least squares mean		Diff.	95% CI for Diff.		*P*
	Present	Absent		Lower	Upper	
1st MTP joint Pain VAS, mm	3.1	6.3	3.2	−6.0	12.4	0.49
Manchester Foot Pain and Disability Index	6.6	5.6	−1.0	−6.9	4.9	0.75
1st MTP joint range of motion, °	66.2	71.2	5.0	−4.8	14.8	0.32
1st MTP joint temperature, °C	25.1	26.2	1.1	0.1	2.1	0.040
Walking velocity, m/s	0.95	1.00	0.05	−0.11	0.21	0.53

Results are presented adjusted for diagnostic group. *Diff.* Difference in mean estimate between presence and absence, *CI* Confidence Interval, *1st MTP joint* first metatarsophalangeal joint, ^o^ degrees

**Table 5:** Association between clinical characteristics and presence of synovitis

	Least squares mean		Diff.	95% CI for Diff.		*P*
	Present	Absent		Lower	Upper	
1st MTP joint Pain VAS, mm	5.7	5.7	0.0	−6.7	6.8	0.99
Manchester Foot Pain and Disability Index	8.4	5.8	−2.7	−6.9	1.6	0.21
1st MTP joint range of motion, °	66.3	71	4.7	−2.5	12.0	0.20
1st MTP joint temperature, °C	26.8	26.2	−0.6	−1.4	0.2	0.13
Walking velocity, m/s	0.94	1.01	0.07	−0.06	0.19	0.29

Results are presented adjusted for diagnostic group. *Diff.* Difference in mean estimate between presence and absence, *CI* Confidence Interval, *1st MTP joint* first metatarsophalangeal joint, ^o^ degrees

## Discussion

This study sought to identify the association between sonographic features of urate deposition, bone erosion and soft tissue inflammation at the 1st MTP joint and clinically-assessed outcomes related to pain, structure and function of the 1st MTP joint while accounting for the diagnosis of the participant as having gout, asymptomatic hyperuricaemia or normouricaemia.

Both sonographic features of MSU deposition, double contour sign and tophus, were associated with increased overall patient-reported foot pain and disability. Previous research has shown that pain and functional limitations related to the feet and lower limbs are a persistent finding in people with gout and asymptomatic hyperuricaemia, even in the absence of acute arthritis [8, 26, 27]. Our findings indicate that urate deposition, rather than soft tissue or bone changes, may be associated with foot-related pain and disability. The lack of association between the double contour sign and other clinical characteristics may be related to the overall low prevalence of the double contour sign in the participants. This may be related to difficulty in differentiating this sign from the normal cartilage interface reflection in addition to the moderate inter-observer reliability [1].

The presence of tophus at the 1st MTP joint was also associated with increased local temperature, which is a clinical feature of underlying inflammation. The presence of larger urate deposits, in the form of tophi, may represent a more progressive disease and an environment more prone to chronic inflammation, and therefore increased temperature, as a result of continuous mechanical stress in the joint during gait. In contrast, no relationship was observed between synovitis (a direct measure of increased soft tissue vascularisation) and increased temperature. It may be that participants with synovitis in the current study had only a mild Doppler signal [1], which may not have been severe enough to be seen in clinical thermal variations. It may be that the relationship between synovitis and temperature would be more pronounced in patients experiencing current acute flares, who experience a higher prevalence of more prominent Doppler signal [28]. This is consistent with studies in rheumatoid arthritis, which have detected sonographic evidence of synovitis despite the absence of any clinical features of inflammation [29–32].

The reduction in walking velocity in those with 1st MTP joint tophus may be a result of the inability to achieve the required 65° of dorsiflexion at the joint necessary for efficient propulsion in combination with reduced 1st MTP joint plantarflexion strength and foot pain [8]. This may support previously proposed hypotheses that altered walking patterns adopted by people with gout are related to pain-avoidance strategies and inefficient 1st MTP joint function [9–11].

The findings from this study should be considered in light of a number of limitations. Firstly, recruitment of participants was undertaken prior to publication of the 2015 ACR/EULAR classification criteria for gout [33] and the majority of the participants with gout were classified based on the 1977 ACR clinical criteria, which has limited specificity [34]. Participants with normouricaemia and hyperuricaemia were classified based on a single measure of serum urate, which may have reduced accuracy compared with multiple measurements over a longer time period. The sampling technique used in the current study resulted in all male participants, meaning the results may not be generalisable to both genders. The adoption of a higher frequency transducer during scanning may have enhanced the ultrasound findings. The cross-sectional design of the study limits the ability to determine whether sonographic features at the 1st MTP joint preceded or were a consequence of the clinical characteristics. Finally, concomitant osteoarthritis, which was not assessed in the current study, is highly prevalent at the 1MTP in people with gout [35], and may contribute to their functional impairment and disability. The current study was limited to sonographic assessment of the 1^st^ MTP joint, and further assessment of other foot and ankle structures for urate deposition, soft tissue and osseous changes, may provide further insight into the impact of sonographic features on overall foot-related pain and disability. Additionally, further longitudinal research may explore the role of foot and ankle structure and function in predicting crystal deposition, inflammation and osseous erosion in individuals with asymptomatic hyperuricaemia.

## Conclusions

In conclusion, this study has shown that sonographic features of MSU deposition, rather than soft tissue inflammation or bone erosion, are associated with clinically evident foot-related pain, impairment and functional disability. This may explain why these individuals with asymptomatic hyperuricaemia, who lacked sonographic features of soft tissue and bone involvement at the 1st MTP joint [1], still presented with high levels of foot pain and disability [8] and exhibited gait impairments [9] when compared with normouricaemic individuals.

## Abbreviations

1st MTP joint: first metatarsophalangeal joint
ARA: American Rheumatism Association
AUT: Auckland University of Technology
BMI: body mass index
MFPDI: Manchester foot pain and disability index
MSU: monosodium urate
ROM: range of motion
VAS: visual analogue scale
